# Automated [^18^F]PSMA-1007 production by a single use cassette-type synthesizer for clinical examination

**DOI:** 10.1186/s41181-020-00101-0

**Published:** 2020-07-29

**Authors:** Sadahiro Naka, Tadashi Watabe, Kenta Kurimoto, Motohide Uemura, Fumihiko Soeda, Oliver C. Neels, Klaus Kopka, Mitsuaki Tatsumi, Hiroki Kato, Norio Nonomura, Eku Shimosegawa, Jens Cardinale, Frederik L. Giesel, Jun Hatazawa

**Affiliations:** 1 Department of Nuclear Medicine and Tracer Kinetics, Osaka University Graduate School of Medicine, 2-2 Yamadaoka, Suita, Osaka, 565-0871 Japan; 2 Department of Radiology, Osaka University Hospital, 2-15 Yamadaoka, Suita, Osaka, 565-0871 Japan; 3 Department of Urology, Osaka University Graduate School of Medicine, 2-2 Yamadaoka, Suita, Osaka, 565-0871 Japan; 4 Institute of Radiopharmaceutical Cancer Research, Helmholtz-Zentrum Dresden-Rossendorf (HZDR), Bautzner Landstraße 400, 01328 Dresden, Germany; 5 Department of Molecular Imaging in Medicine, Osaka University Graduate School of Medicine, 2-2 Yamadaoka, Suita, Osaka, 565-0871 Japan; 6 Department for Nuclear Medicine, University Hospital Heidelberg, INF 400, 69120 Heidelberg, Germany; 7 Department of Quantum Cancer Therapy, Research Center for Nuclear Physics, Osaka University, 10-1 Mihogaoka, Osaka, Ibaraki 567-0047 Japan

**Keywords:** PET, PSMA, [^18^F]PSMA-1007, Cassette-type radiosynthesizer, SPE

## Abstract

**Background:**

[^18^F]PSMA-1007, a positron emission tomography (PET) tracer, specifically targets prostate-specific membrane antigen (PSMA), which is highly expressed in prostate cancer. PSMA-PET is effective especially for regional detection of biochemical recurrence, which significantly affects patient management. Herein, we established and optimized a one-step radiolabeling protocol to separate and purify [^18^F]PSMA-1007 with a CFN-MPS200 synthesizer for clinical application.

**Results:**

A dedicated single use cassette and synthesis program for [^18^F]PSMA-1007 was generated using a single-step method for direct precursor radiolabeling. In the cassette, three tube types (fluoro-elastomer, PharMed® BPT, silicone) and two different precursor salts (trifluoroacetic acid or acetic acid) were compared for optimization. Furthermore, three-lot tests were performed under optimized conditions for quality confirmation. Activity yields and mean radiochemical purity of [^18^F]PSMA-1007 were > 5000 MBq and 95%, respectively, at the end of synthesis, and the decay-corrected mean radiochemical yield from all three cassettes was approximately 40% using a trifluoroacetic acid salt precursor. Fluoro-elastomer tubings significantly increased the amount of non-radioactive PSMA-1007 (8.5 ± 3.1 μg/mL) compared to those with other tubings (0.3 μg/mL). This reduced the molar activity of [^18^F]PSMA-1007 synthesized in the cassette assembled by fluoro-elastomer tubings (46 GBq/μmol) compared to that with PharMed® BPT and silicone tubings (1184 and 1411 GBq/μmol, respectively). Residual tetrabutylammonium, acetonitrile, and dimethyl sulfoxide levels were <  2.6 μg/mL, < 8 ppm, and <  11 ppm, respectively, and ethanol content was 8.0–8.1% in all three cassettes and two different salts. Higher activity yields, radiochemical purities, and decay-corrected radiochemical yields were obtained using an acetic acid salt precursor rather than a trifluoroacetic acid salt precursor (7906 ± 1216 MBq, 97% ± 0%, and 56% ± 4%). In the three-lot tests under conditions optimized with silicone cassettes and acetic acid salt precursor, all quality items passed the specifications required for human use.

**Conclusions:**

We successfully automated the production of [^18^F]PSMA-1007 for clinical use and optimized synthesis procedures with a CFN-MPS200 synthesizer using a silicone cassette and acetic acid salt precursor. Cassette availability will facilitate a wide spread use of [^18^F]PSMA-1007-PET, leading to an effective prostate cancer management.

## Background

The prostate-specific membrane antigen (PSMA) is a major target for theranostics in prostate cancer (Israeli et al. 1994; Herrmann et al. 2015; Ferdinandus et al. 2018). Positron emission tomography (PET) tracers labeled with ^68^Ga such as [^68^Ga]Ga-PSMA-11 have been used for diagnosis (Eder et al. 2012; Afshar-Oromieh et al. 2012; Rowe et al. 2016). ^68^Ge/^68^Ga generators enable PET examination at remote facilities; however, they are associated with logistical limitations such as a long delivery duration due to shortage of commercially available approved generators; additionally, high activity yields cannot be obtained due to capacity limitations of the available generators. In addition, ^68^Ga production by a cyclotron has been used by some PET facilities for clinical use; however, this strategy is not popular. In recent years, several ^18^F-labeled PET tracers targeting PSMA have been developed, exemplified by [^18^F] DCFBC, [^18^F] DCFPyL, and [^18^F]PSMA-1007 (Chen et al. 2011; Mease et al. 2008; Cardinale et al. 2017a). Amongst them, [^18^F]PSMA-1007 has especially attracted attention for its minimal urinary excretion, which enables better clinical interpretation (Cardinale et al. 2017a; Giesel et al. 2017; Giesel et al. 2016; Giesel et al. 2019).

Radiosynthesis of [^18^F]PSMA-1007 via a two-step radiolabeling method using 6-[^18^F]fluoropyridine-3-carboxylic acid ([^18^F]F-Py-TFP) has previously been conducted (Cardinale et al. 2017a). However, this method was unsuitable for routine clinical examination since the synthesis procedure was complicated and resulted in rather poor radiochemical yields (the radiochemical yield was 1.5–6.0%). Subsequently, a single-step method to directly radiolabel a quaternary ammonium salt precursor using [^18^F] fluoride in a nucleophilic substitution reaction and a solid phase extraction (SPE) cartridge column instead of high-performance liquid chromatography (HPLC) to separate and formulate [^18^F]PSMA-1007 has been developed (Cardinale et al. 2017b). As a result, the [^18^F]PSMA-1007 synthesis procedure was simplified and the radiochemical yield was greatly improved. Further, the authors had optimized the protocols of various commercially available synthesizers (Tracerlab FX FN and TRACERlab MX (GE Healthcare), NEPTIS mosaic-RS (ORA) and SYNTHERA+ (IBA)) (Cardinale et al. 2017b). However, the production ability of CFN-MPS200 synthesizer (Sumitomo Heavy Industries, Tokyo, Japan), which has been installed primarily in various Asian countries including Japan, has not yet been investigated. In Asia, the prevalence of prostate cancer has increased rapidly in the past years (Chen and Ren 2014) and the World Health Organization has reported that the number of cases would nearly double by 2030 (GLOBOCAN, 2012). Furthermore, PSMA-PET is effective especially for the regional detection of biochemical recurrence and has a significant influence on patient management (Giesel et al. 2019; Koerber et al. 2018). For this reason, [^18^F]PSMA-1007 PET examination is expected to be performed on more patients for the precise evaluation of prostate cancer, making its stable and reproducible production to be essential. In this study, we aimed to determine the optimal materials for cassette assembly and precursor salts, and optimize a one-step radiolabeling method, as well as an SPE method, for the separation and formulation of [^18^F]PSMA-1007 using a CFN-MPS200 synthesizer. We also verified its quality and application for clinical examination with three-lot tests.

## Methods

### Reagents and SPE columns

**Fig. 1:**
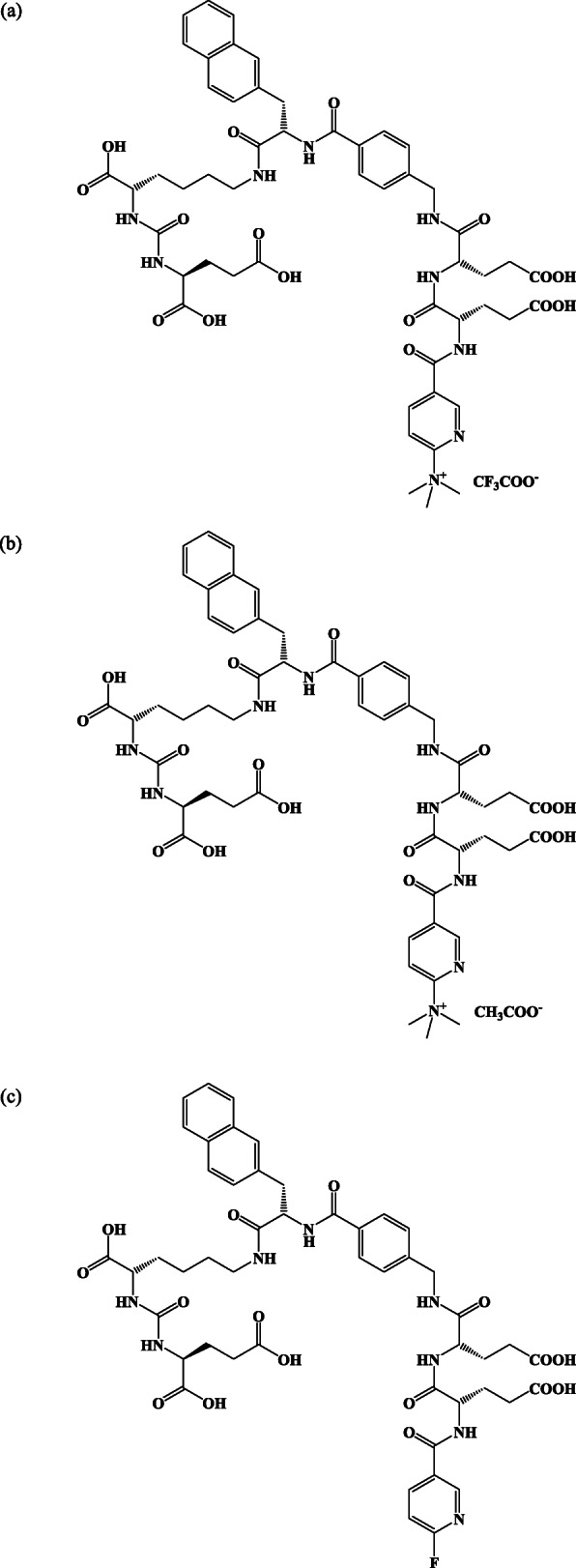
Structures of the two types of precursors and the reference standard of [^18^F]PSMA-1007. The precursor for direct radiolabeling was trifluoroacetic acid (TFA) salt (**a**) and acetic acid (ACE) salt (**b**) of the precursor compound bearing tri-methyl ammonium as the leaving group. The reference standard of [^18^F]PSMA-1007 is shown (**c**)

Two types of precursors, with manufacturer certified stability and a reference standard of [^18^F]PSMA-1007 (Fig. 1), were provided by ABX (Radeberg, Germany). Tetrabutylammonium hydrogen carbonate (0.075 M, TBAHCO_3_) aqueous solution, stabilized with ethanol as an eluent of [^18^F] fluoride, was also purchased from the same supplier. The other reagents for [^18^F]PSMA-1007 synthesis were selected based on the grade as follows: acetonitrile (MeCN) for DNA synthesis (Merck, Darmstadt, Germany), super dehydrated dimethyl sulfoxide (DMSO; FUJIFILM Wako Pure Chemical, Osaka, Japan), and ethanol (Merck), sodium ascorbate (Spectrum, New Brunswick, NJ), water for injection (WFI), and isotonic sodium chloride solution (saline) (Otsuka Pharmaceutical, Tokyo, Japan) of Pharmacopeia grade. All reagents were used as provided by commercial sources and were not additionally purified. Sep-Pak® Light Cartridge Accell™ Plus QMA carbonate for the collection of [^18^F] fluoride, as well as CHROMAFIX® PS-H^+^ (L) and CHROMAFIX® C_18ec_ (M) for the purification of [^18^F]PSMA-1007, were purchased from Waters (Milford, MA) and Machery-Nagel (Düren, Germany), respectively.

### Cassette for synthesis and other materials

**Fig. 2:**
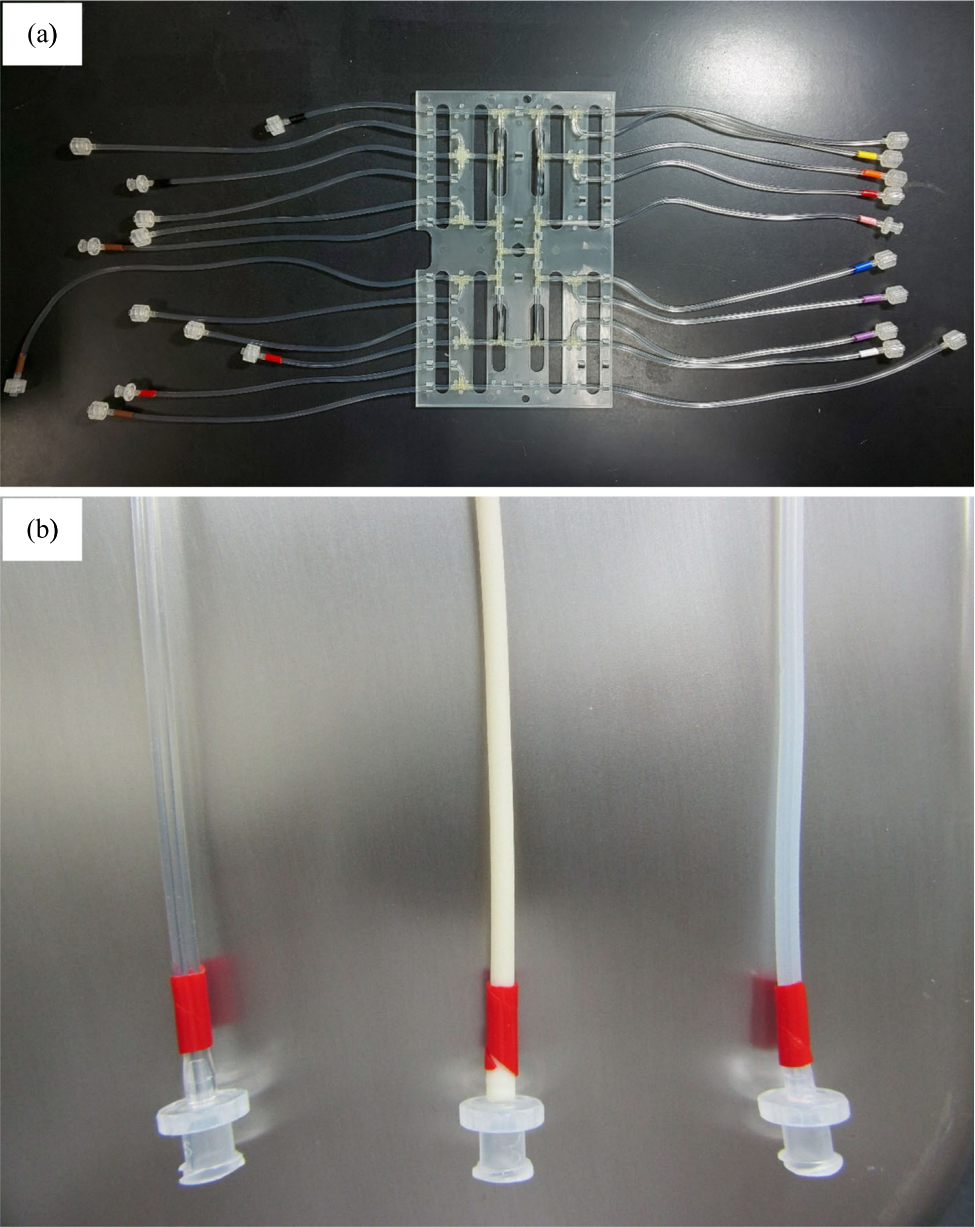
The cassette materials for the synthesis of [^18^F]PSMA-1007 solution. **a** The [^18^F] fluoroethyl choline cassette as a commercial item provided by Sumitomo Heavy Industries. **b** The left clear tube is the Fluoro-elastomer, the middle white color tube is the PharMed® BPT, and the right translucent tube is silicone

The single use [^18^F]PSMA-1007 cassette was a modified [^18^F] fluoroethyl choline cassette purchased as a commercial item from Sumitomo Heavy Industries. Three different types of tubing material for the cassette were compared (Fig. 2). One material (fluoro-elastomer) was a bilayer tube made of fluoro-elastomer and fluoro-polymer layers for excellent chemical resistance to organic solvents. Fluoro-elastomer has high chemical resistance to various organic solvents, and the tubings of the commercially available [^18^F] fluoroethyl choline cassette are made of this material. Another tubing was made of thermoplastic elastomer (PharMed® BPT); this tube is protected from acid, alkalinity, and oxidation. The final material type selected was silicone, as a tube with low cost and high versatility. The reaction vessel used 10-mL syringe-vials (Nichiden-Rika Glass, Hyogo, Japan) and the other materials were purchased as commercial items.

### Production of [^18^F]PSMA-1007 solution with the synthesizer

**Fig. 3:**
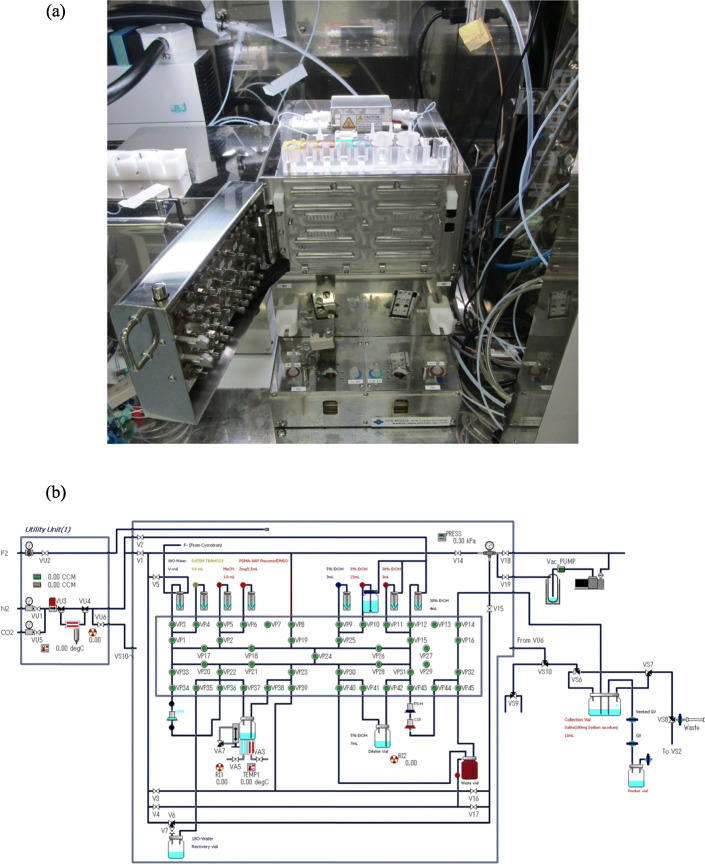
Diagram of the synthesis of [^18^F]PSMA-1007 injection. **a** A single use cassette type synthesizer. **b** Cupid program for [^18^F]PSMA-1007 synthesis

[^18^F] Fluoride was produced by the ^18^O(p, n)^18^F nuclear reaction with proton irradiation to 2.3 mL [^18^O]H_2_O (> 98 atom %, Rotem, Mishor Yamin, Israel) using a cyclotron with 18 MeV energy (CYPRIS HM-18, Sumitomo Heavy Industries). The synthesis of [^18^F]PSMA-1007 was performed using a single use cassette-type CFN-MPS200 synthesizer, and synthesis procedures were controlled by the Cupid System (Sumitomo Heavy Industries, Fig. 3). The vacuum of the synthesizer was set to − 100 kPa and controlled by the on/off function of the “VAC pump” on the Cupid System. The gas for synthesis was N_2_ (> 99.9999%, Taiyo Nippon Sanso, Tokyo, Japan), and the flow rate was set with the Cupid System in each step.

**Fig. 4:**
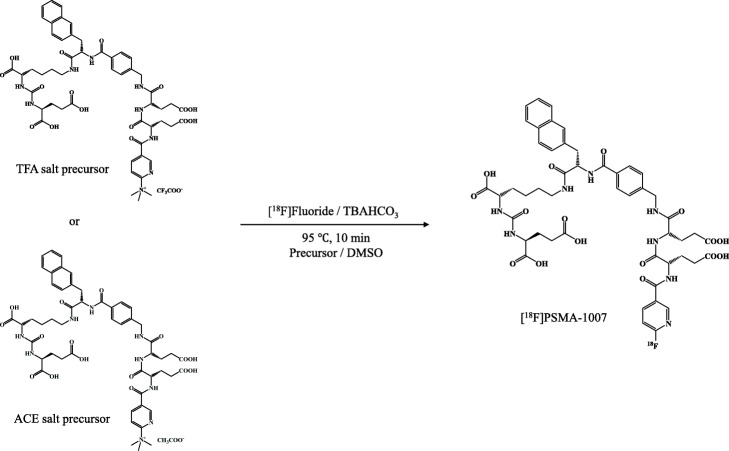
Pathway of the radiosynthesis of [^18^F]PSMA-1007. The precursor trifluoroacetic acid (TFA) salt was used at 3 mg in 2 mL of DMSO and the precursor acetic acid (ACE) salt was used at 2 mg in 2 mL of DMSO. The other conditions were the same with both precursors

[^18^F] Fluoride was collected into a 5 mL V-shaped vial (Wheaton, Millville, NJ) connected to valve VP3 after bombardment of [^18^O]H_2_O at 25 μA for 15 min and then loaded onto a QMA carbonate column (pretreated with 40 mL of WFI) via VP3 with the vacuum and 50 mL/min N_2_ gas and discarded to ^18^O-water recovery vial via VP35. After trapping on QMA carbonate, [^18^F] fluoride was eluted with 0.6 mL of 0.075 M TBAHCO_3_ aqueous solution (yellow holder) into a reaction vessel via VP4 to VP37 using a vacuum. The eluent was eliminated at 120 °C with a vacuum and 50 to 80 mL/min N_2_ gas flow. Further, 1 mL of MeCN in a 3 mL mini-vial (MV-1, Nichiden-Rika Glass, orange holder) for azeotropic drying was fed into a reaction vessel via VP5 to VP22 and VP37 and completely dried at 120 °C with a vacuum and 40 mL/min N_2_ gas flow. Then, 2 mL of DMSO containing 3 mg trifluoroacetic acid (TFA) salt precursor or 2 mg acetic acid (ACE) salt precursor in MV-1 (red holder) was added into the reaction vessel via VP6 to VP22 and VP37, and radio-fluorination was performed at 95 °C for 10 min with a closed system (Fig. 4). The reaction solution was transferred to a dilution vial (20 mL sealable sterile vial, Mita Rika Kogyo, Hyogo, Japan) containing 7 mL of 5% ethanol solution via VP38 to VP28 and VP42 and mixed well using 200 mL/min N_2_ gas flow. Then, the path to the dilution vial (VP9 to VP24 and VP42) was washed with 3 mL of 5% ethanol solution in MV-1 (blue holder) and mixed again using 200 mL/min N_2_ gas flow. The mixture (total volume 12 mL) was passed through the PS-H^+^ and C_18ec_ columns (PS-H^+^ on top, C_18ec_ bottom, both pretreated with 3 mL ethanol and 25 mL of 5% ethanol solution after connecting both columns) via VP42 and was discarded to a waste vial via VP45. Next, both columns were washed with 23 mL of 5% ethanol from a 30 mL sealable sterile vial (Mita Rika Kogyo, left side purple holder) via VP10, followed by 3 mL of 30% ethanol in MV-1 (right side purple holder) via VP11 and was discarded to a waste vial via VP45. [^18^F]PSMA-1007 were eluted using 4 mL of 30% ethanol in a 5 mL V-shaped vial (Wheaton, no-color holder) from the SPE columns into the collection vial (30-mL sealable sterile vial) containing 100 mg of sodium ascorbate in 11 mL of saline via VP12 to VP14. The process of loading the reaction mixture onto the column and transfer to a collection vial was performed with a 50 mL/min N_2_ gas flow, whereas column washing was performed with a vacuum and 50 mL/min N_2_ gas flow. In the cassette, a 22-G needle was used to transfer and discard the liquid from the vial and to supply and exhaust the N_2_ gas. For the three-lot tests, the [^18^F]PSMA-1007 injection solution in the collection vial was transferred to a product vial (20-mL sealable sterile vial, Mita Rika Kogyo) through the sterile 0.22-μm Millex GV filter (Merck Millipore, Burlington, MA) with 50 mL/min N_2_ gas flow for sterility.

### Quality control for [^18^F]PSMA-1007 injection

The activity yields of [^18^F]PSMA-1007 and the parts of the synthesizer (columns, reaction vessel, vials, cassette and waste liquid.), relevant for the calculated radiochemical yield, were measured by a dose-calibrator. The measurements of radiochemical purity, PSMA-1007 content, and chemical impurities were performed in accordance with a previous report, with modification (Cardinale et al. 2017b). Namely, these items were analyzed with an SPD-20A ultraviolet detector (wavelength 254 nm) with a Shimadzu HPLC system and Gabi-Star radioactivity detector (Raytest) using a Chromolith performance RP-18e 100 mm × 4.6 mm column (Merck). The mobile phase was MeCN (solvent A) and 0.1% TFA (solvent B) for the gradient (the gradient condition is shown as follows: A 5% to 15% from 0 to 1.5 min; A 15% to 35% from 1.5 to 10.5 min; A 35% to 95% from 10.5 to 13 min; A 95% to 5% from 13 to 19 min), the flow rate was 3.0 mL/min, and the column oven was set to 30 °C. The residual tetrabutylammonium (TBA) was analyzed with a conductivity detector (CDD10A_VP_) with a Shimadzu HPLC system using an IC YS-50125 × 4.6 mm column (Showa Denko, Tokyo, Japan). The mobile phase was 4 mmol/L nitric acid/MeCN (65/35), the flow rate was 1.5 mL/min, and the column oven was set to 40 °C. The residual MeCN, DMSO, and ethanol concentrations were analyzed with a flame ionization detector with a Shimadzu gas chromatograph system GC-2014 using a DB-624 30 m × 0.32 mm, 1.8 μm column (Agilent, Santa Clara, CA) and split injection mode (split ratio of 30:1). The flow of helium, as a carrier gas, was 2 mL/min, and the column, injector, and detector temperatures were 40 °C for 5 min to 200 °C (20 °C/min) for 3 min, 200 °C, and 250 °C, respectively. The activity yields, radiochemical yield, radiochemical purity, [^18^F]PSMA-1007 M activity, chemical impurities, residual TBA, residual MeCN, DMSO, and ethanol were compared between three types of cassettes using TFA salt precursor and silicone cassette using the ACE salt precursor.

For three-lot tests, the appearance, particles, identity, half-life, activity yields, concentration, radionuclidic identity, radionuclidic purity, pH, sterility, endotoxin, and filter integrity, in addition to the aforementioned comparable items, were assessed. The quality control standards referred to the [^18^F]PSMA-1007 draft monograph (3116) in the European Pharmacopoeia. However, the endotoxin was changed to < 15.0 EU/mL in accordance with the standard setting procedure in the Japanese Pharmacopoeia. HPLC conditions for the measurements of radiochemical purity, content of PSMA-1007, and chemical impurities were changed based on the conditions offered by ABX. The column used was the Ascentis Express Peptide Es-C18 150 mm × 4.6 mm column (SUPELCO, Bellefonte, PA). The mobile phase was 20 mM phosphate buffer, pH 2.5 (solvent A) and MeCN (solvent B) for the gradient (the gradient condition was as follows: A 77% from 0 to 2.0 min; A 77% to 70% from 2.0 to 14.0 min; A 70% to 40% from 14.0 to 17.0 min; A 40% from 17.0 to 21.0 min). The flow rate was 1.3 mL/min, and the column oven and wavelength were set to 30 °C and 225 nm, respectively. The TBA analysis was also changed to the TLC method according to a previous report (Cardinale et al. 2017b).

pH was measured using a F-72 pH/ion meter and Micro ToupH Electrode for low-volume solutions (HORIBA, Kyoto, Japan) calibrated with pH standard solutions, and the endotoxin test was performed with the Toxinometer® ET-6000 (FUJIFILM Wako Pure Chemical) according to the Japanese Pharmacopeia. During the sterility test, 0.5 mL of [^18^F]PSMA-1007 solution was directly injected into tryptic soy broth and thioglycolate liquid medium (Merck) and incubated at 22.5 °C and 32.5 °C for 14 days, respectively. At intervals during the incubation period (two times) and at the final day, we examined the medium for macroscopic microbial growth. The bubble point test, as filter integrity, was performed with a UG-FT02 automatic filter integrity tester (Universal Giken, Kanagawa, Japan) and the specification of the bubble point (> 286 kPa), was applied based on a preliminary test using 5 to 10% ethanol solution.

### Clinical research based on a prostate Cancer patient

After obtaining approval from the institutional review board of Osaka University Hospital and informed consent from a prostate cancer patient before therapy, PSMA-PET was performed 60 min following the intravenous injection of [^18^F]PSMA-1007 (193 MBq) as a proof-of-concept study.

### Statistical analysis

Microsoft Excel 2013 (Microsoft Corp., Redmond, WA) was used for the statistical analyses. Comparisons between two groups were performed based on an unpaired t-test. *P* < 0.05 was considered indicative of a statistically significant difference.

## Results

**Fig. 5:**
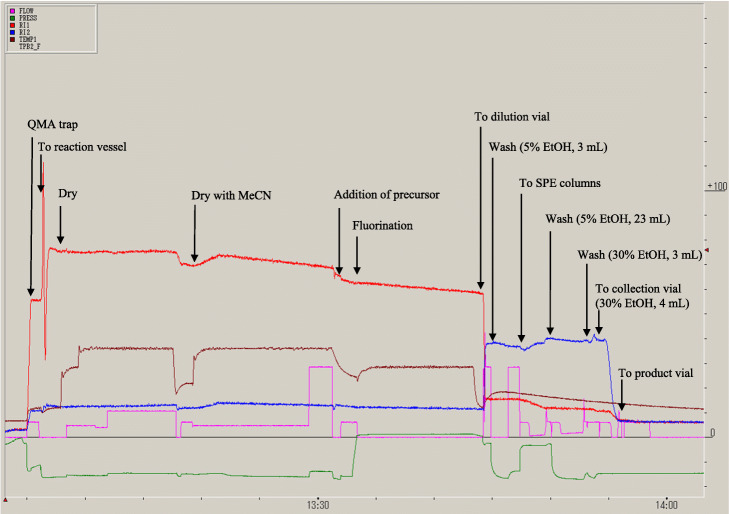
The typical trend graph of [^18^F]PSMA-1007 synthesis. Total synthesis time was 62 min on average. RI1 was installed via the reaction vessel (red line) and RI2 was installed via the dilution vial and solid phase extraction (SPE) columns (blue line)

**Fig. 6:**
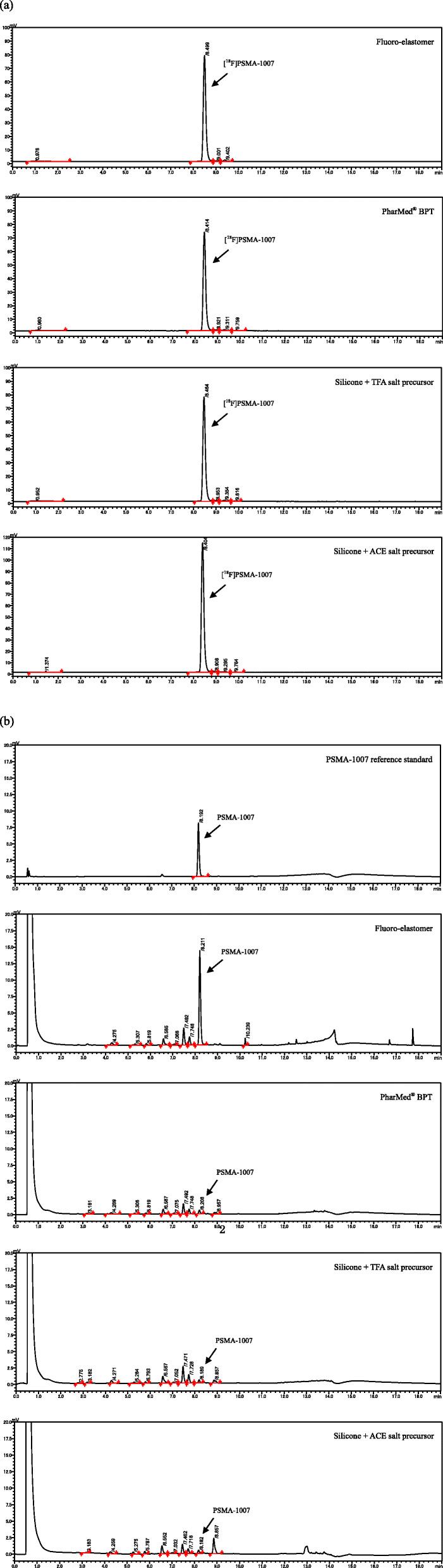
Chromatograms of [^18^F]PSMA-1007. **a** The typical radio-chromatogram of [^18^F]PSMA-1007 solution using cassettes with three types of tubing material. The radiochemical purity was 95% with each cassette with trifluoroacetic acid (TFA) salt precursor. For the acetic acid ACE salt precursor, the radiochemical purity was > 97%. **b** The typical UV-chromatogram of the PSMA-1007 reference standard and [^18^F]PSMA-1007 solution produced using cassettes with three types of tubing material with precursors. The PSMA-1007 reference standard was 5 μg/mL (retention time (RT) 8.192 min). The PSMA-1007 with the Fluoro-elastomer was 8.5 μg/mL (RT 8.211 min), whereas that obtained with the other tubing material was 0.3 μg/mL (RT 8.208 and 8.189 min). The highest single chemical impurity peak was detected at approximately 7.5 min of retention time and the impurity profiles of the chromatograms were similar. For the ACE salt precursor, the highest single chemical impurity peak was detected at a retention time of 8.9 min PnarMed® BPT

The production of [^18^F]PSMA-1007 injection solution was completed within 62 min on average including the trapping of [^18^F] fluoride from the V-vial to the QMA column on the synthesizer (Fig. 5). The results of [^18^F]PSMA-1007 automated synthesis using a single-use cassette with three different types of tubing and the two precursors are shown in Tables 1 and 2. For all cassettes, the activity yields at the end of synthesis (EOS) were > 5000 MBq. For the TFA salt precursor, the radiochemical yield calculated from total activity yields of the parts for synthesis was 42% ± 4%. The radiochemical purity was 95% and three radiochemical impurities were detected using fluoro-elastomer; in addition, radiochemical impurities in silicone and PharMed® BPT were also detected at 9.8 min (Fig. 6a). The PSMA-1007 concentration using Fluoro-elastomer was 8.5 ± 3.1 μg/mL, which was much higher than that with the tubing material (0.3 μg/mL with PharMed® BPT and silicone tubings, Fig. 6b). The molar activities calculated from these results were 46, 1184 and 1411 GBq/μmol, respectively. With respect to the chemical impurities, the highest values were 1.5, 1.0, and 1.4 μg/mL and these peaks were detected at approximately 7.5 min retention time (Fig. 6b). Total chemical impurities without PSMA-1007 were 4.1, 3.0, and 4.2 μg/mL, and the impurity profiles of the chromatograms were similar between PharMed® BPT and silicone cassettes (Fig. 6b).

**Table 1:** Comparison of the three types of cassettes

Cassette material	Fluoro- elastomer (*n* = 3)	PharMed® BPT (*n* = 3)	Silicone (*n* = 3)	Silicone (*n* = 7)
Precursor form	TFA salt	TFA salt	TFA salt	ACE salt
Activity yields (MBq)	5437 ± 504 *	5353 ± 273 *	5507 ± 115 *	7906 ± 1216 *
Radiochemical yield (%)	43 ± 7	41 ± 1 *	41 ± 1 *	56 ± 4 *
Radiochemical purity (%)	95 ± 1 *	95 ± 0 *	95 ± 0 *	97 ± 0 *
PSMA-1007 (μg/mL)	8.5 ± 3.1 *	0.3 ± 0.0	0.3 ± 0.0 *	0.4 ± 0.0 *
Molar activity (GBq/μmol)	46 ± 19 *	1184 ± 161	1411 ± 169	1463 ± 189 *
Highest single chemical impurity (μg/mL)	1.5 ± 0.2	1.0 ± 0.0	1.4 ± 0.2	1.2 ± 0.3
Total chemical impurities (μg/mL)	4.1 ± 0.4	3.0 ± 0.6	4.2 ± 0.3	4.1 ± 0.7

*P < 0.05 vs Silicone, ACE salt

Activity yields, radiochemical yield, radiochemical purity, PSMA-1007, molar activity, highest single chemical impurity, and total chemical impurities were compared among the three types of cassettes using precursor trifluoroacetic acid (TFA) salt and silicone cassette using acetic acid (ACE) salt precursor (*p* < 0.05 by unpaired t-test compared to silicone, precursor of ACE salt)

**Table 2:** Comparison of residual TBA, MeCN, DMSO, and ethanol among three cassette types

Cassette material	Fluoro-elastomer (*n* = 3)	PharMed® BPT (*n* = 3)	Silicone (*n* = 3)	Silicone (*n* = 7)
Precursor form	TFA salt	TFA salt	TFA salt	ACE salt
Residual TBA (μg/mL)	< 2.6	< 2.6	< 2.6	< 2.6
Residual MeCN (ppm)	< 8	< 8	< 8	< 8
Residual DMSO (ppm)	< 11	< 11	< 11	< 11
Ethanol (v/v %)	8.1 ± 0.2	8.0 ± 0.1	8.0 ± 0.2	7.8 ± 0.1

**Fig. 7:**
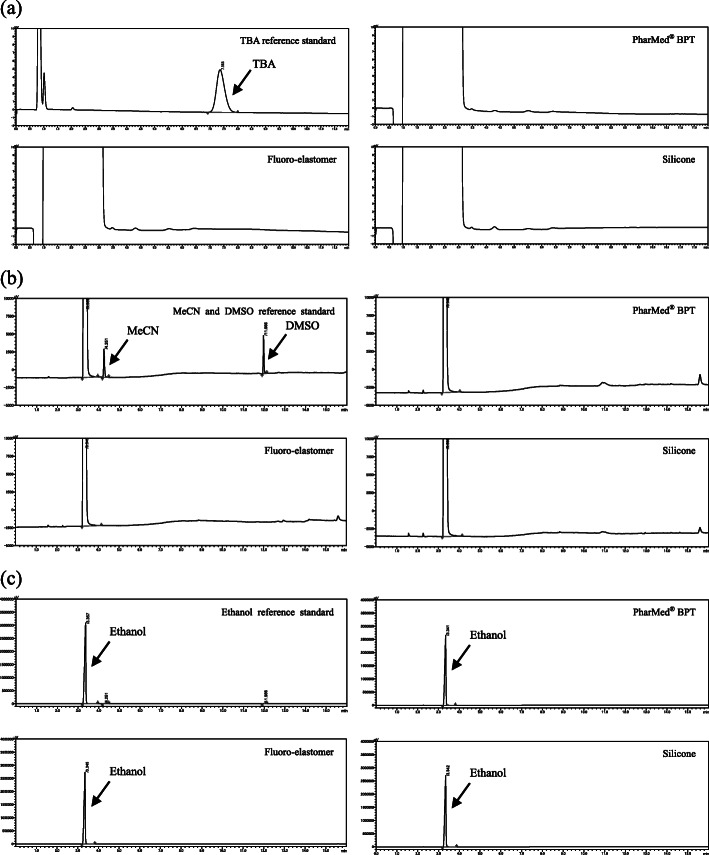
Chromatograms of reference standards and [^18^F]PSMA-1007. **a** The typical ion-chromatogram of the tetrabutylammonium (TBA) reference standard and [^18^F]PSMA-1007 solution produced using cassettes with three types of tubing material with the TFA salt precursor. The TBA reference standard was 260 μg/mL (RT 7.363 min). The TBA peak of [^18^F]PSMA-1007 solution was not detected. The lower limit of the calibration curve was 2.6 μg/mL. **b** The typical gas-chromatogram of the acetonitrile (MeCN) and DMSO reference standards and [^18^F]PSMA-1007 solution produced using cassettes with three types of tubing material with the TFA salt precursor. The MeCN and DMSO reference standards were 79 ppm (RT 4.251 min) and 110 ppm (RT 11.966 min), respectively. The MeCN and DMSO peaks of the [^18^F]PSMA-1007 solution were not detected. The MeCN and DMSO lower limit of the calibration curves were 8 ppm and 11 ppm, respectively. **c** The typical gas-chromatogram of the ethanol reference standard and [^18^F]PSMA-1007 solution produced using three types of cassettes with the TFA salt precursor (this chromatogram is a zoom out of Fig. 7b). The ethanol reference standard was 10% v/v (RT 3.357 min). The ethanol peak of the [^18^F]PSMA-1007 solution was detected at approximately 3.4 min of retention time and the concentration was approximately 8% v/v in all cassettes

For all cassettes, the residual TBA, MeCN, and DMSO concentrations were less than the lower limit of the calibration curve, specifically < 2.6 μg/mL, < 8 ppm, and <  11 ppm, respectively (these peaks were not detected; Fig. 7a, b). The ethanol content was 8.0% to 8.1% v/v. This result was reflected by the amount of ethanol (8.0% v/v) for the [^18^F]PSMA-1007 solution (Fig. 7c).

[^18^F]PSMA-1007 was produced using ACE salt precursor in a single-use cassette with silicone tubings, and the total activity yield was 7906 ± 1216 MBq and the radiochemical yield was 56% ± 4%. The radiochemical purity was 97% ± 0%, and the highest value of the chemical impurity in the [^18^F]PSMA-1007 solution was detected at approximately 8.9 min of retention time. The results of residual TBA, MeCN, DMSO, and ethanol concentrations were the same as those with the TFA salt precursor using the cassette with silicone tubings.

For the three-lot tests, the results of quality control tests of the [^18^F]PSMA-1007 solution using the cassette with silicone tubings and ACE salt precursor are shown in Table 3. The activity yields obtained were > 7000 MBq following sterile filtration. The radiochemical purity was > 97% at EOS, and stability was confirmed until 6 h after EOS (> 95%) by HPLC. [^18^F] Fluoride was determined by radio-TLC (< 5%, data not shown). The molar activity calculated from the activity yields at EOS and PSMA-1007 (0.5–0.8 μg/mL) was 980 ± 198 GBq/μmol. The residual TBA, MeCN, and DMSO concentrations were <  26 μg/mL, < 10 ppm, and <  138 ppm (less than the limit of quantitation determined in the validation), respectively. The pH was 6.6 to 6.7 and the bubble point was 317 to 327 kPa. In addition, all other quality control items would have passed specifications based on the European Pharmacopeia (Cardinale et al. 2017b).

**Table 3:** Results of synthesis and quality control tests for [^18^F]PSMA-1007 injection solution

Test items	Acceptance criteria	Lot No.1	Lot No.2	Lot No.3
Activity yields (MBq)	-	7,580	8,570	7,690
Appearance	Clear and colorless	Clear and colorless	Clear and colorless	Clear and colorless
Particle	None	None	None	None
Identity of [^18^F]PSMA-1007 (min)	RT of PSMA-1007 + 0.3–+ 0.7	+ 0.4	+ 0.4	+ 0.4
Half-life (min)	105–115	110	110	109
Concentration of activity yields (MBq/mL)	> 18.5	610.7	667.5	621.5
Ethanol (v/v %)	< 10.0	7.8	7.9	7.9
Residual MeCN (ppm)	< 410	< 10	< 10	< 10
Residual DMSO (ppm)	< 5,000	< 138	< 138	< 138
PSMA-1007 (μg/mL)	< 10	0.5	0.8	0.7
(Molar activity (GBq/μmol))	-	1,204	829	907
Highest single chemical impurity (μg/mL)	< 10	0.7	0.9	1.0
Total chemical impurities + PSMA-1007 (μg/mL)	< 50	2.7	3.1	3.2
TBA (μg/mL)	< 260	< 26	< 26	< 26
Radionuclidic identity	Exhibits the peak at 511 keV	Exhibits the peak at 511 keV	Exhibits the peak at 511 keV	Exhibits the peak at 511 keV
Radiochemical purity (at EOS) (%)	> 95	98	97	98
[^18^F]Fluoride (at EOS) (%)	< 5	< 5	< 5	< 5
Radiochemical purity (6 h after EOS)	> 95	97	97	98
[^18^F]Fluoride (6 h after EOS)	< 5	< 5	< 5	< 5
Radionuclidic purity	Exhibits no peak except 511 keV and 1022 keV	Exhibits no peak except 511 keV and 1022 keV	Exhibits no peak except 511 keV and 1022 keV	Exhibits no peak except 511 keV and 1022 keV
pH	4.5–8.5	6.6	6.7	6.7
Sterility	Sterile	Sterile	Sterile	Sterile
Endotoxin (EU/mL)	< 15.0	< 2.0	< 2.0	< 2.0
Filter integrity test (kPa)	> 286	318	317	327

*EOS* end of synthesis

*RT* retention time

**Fig. 8:**
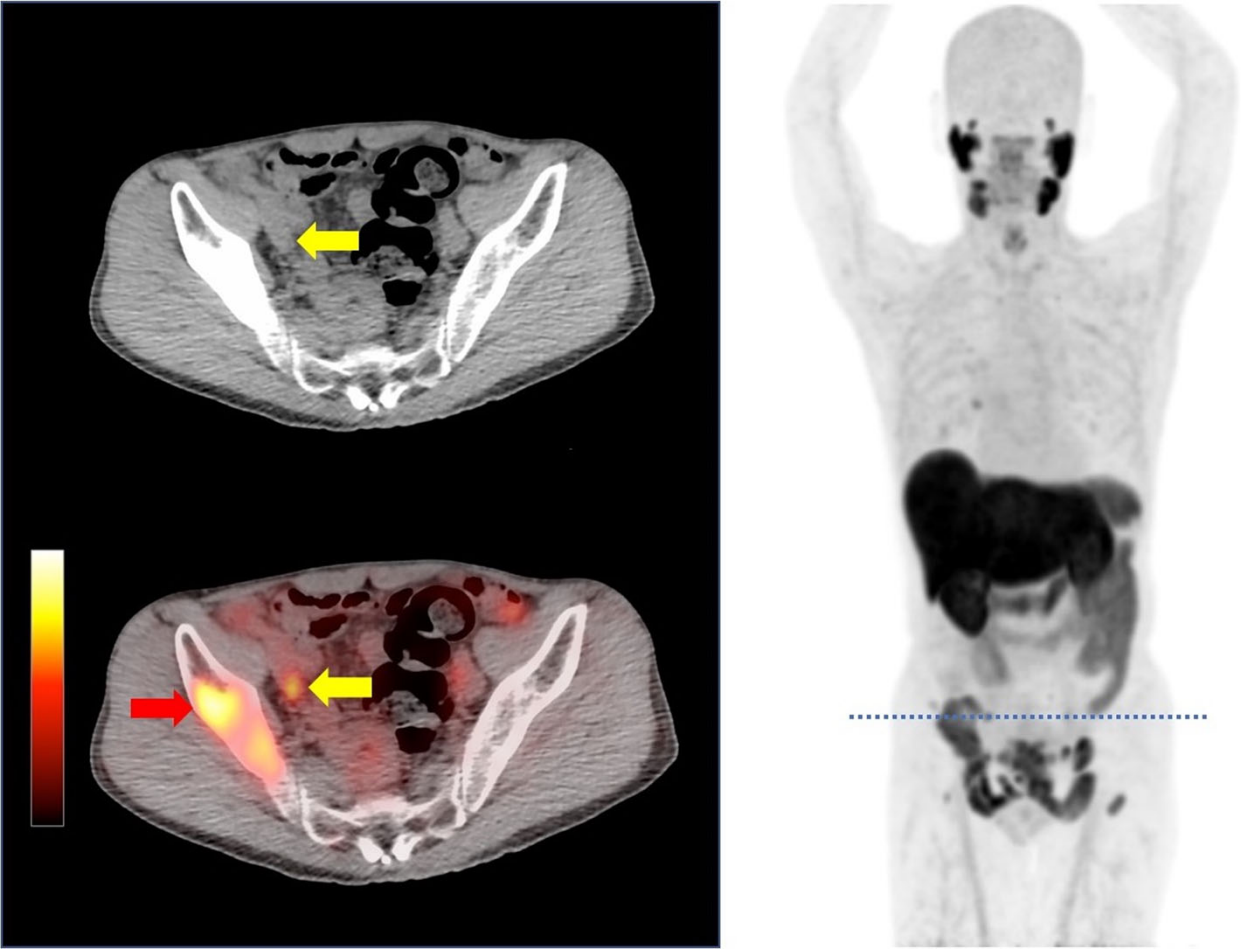
[^18^F]PSMA-1007 PET/CT images of a metastatic prostate cancer patient. A dotted cross section of whole-body PET (right) is displayed on the CT and PET/CT image (left). In addition to right iliac metastasis (red arrow), we observed a small lymph node metastatic lesion (yellow arrows), which would be virtually impossible to detect by conventional CT imaging

[^18^F]PSMA-1007 PET/CT images of a metastatic prostate cancer patient are shown in Fig. 8 as proof-of-concept using one of the formulations synthesized at Osaka University hospital. High uptakes were clearly detected in iliac lymph node and bone metastases.

## Discussion

In this study, we have achieved the automated production of [^18^F]PSMA-1007 injection solution for clinical examination with a new cassette and optimized the synthesis procedure using a CFN-MPS200 synthesizer. We successfully obtained [^18^F]PSMA-1007 with a high radiochemical yield and high molar activity, which would make it possible to scan up to 9 patients (from a single batch, assuming the patient had an average body weight of 60 kg), with a [^18^F]PSMA-1007 injection dose of 3.7 MBq/kg at 30 min intervals using only one PET-CT scanner. In this study, we were able to obtain sufficient activity yields for [^18^F]PSMA-1007 via a 15 min irradiation. Through this approach, we would be able to get higher activity yields of [^18^F]PSMA-1007 and examine more patients by extending the irradiation time, in other words, using higher amounts of starting [^18^F]fluoride. The [^18^F] FEC cassette could be used as the platform for the [^18^F]PSMA-1007 cassette, and the activity yields, radiochemical yield, and radiochemical purity were the same among the three types of tubing materials. However, the material has to be changed from Fluoro-elastomer to PharMed® BPT or silicone, because the molar activity of [^18^F]PSMA-1007 was 28-fold higher with the latter. We have previously reported that the molar activity or ligand concentration will affect tumor uptake and physiological accumulation (Soeda et al. 2019). In other words, the very low molar activity using Fluoro-elastomer tubing material could potentially decrease the accumulation of [^18^F]PSMA-1007 in the tumor, which potentially would result in a decrease in lesion detection. It was inferred that the elution of fluorine from the inner wall of the Fluoro-elastomer using a pinch valve influences the production of a large quantity of non-radioactive PSMA-1007. However, the quantity of non-radioactive PSMA-1007 with the PharMed® BPT and silicone was very small (0.3 to 0.4 μg/mL) and this result is sufficient based on specifications of the draft monograph for [^18^F]PSMA-1007 in the European Pharmacopeia (this specification is < 100 μg/V, V was chosen with 10 mL per patient as a maximum volume of injection solution). In addition, the molar activity was > 1000 GBq/μmol, approximately 10-fold higher than that reported previously (126 ± 42 GBq/μmol) (Cardinale et al. 2017b). Furthermore, in analogy to the [^18^F] FET monograph (No. 07/2015:2466 Eur. Ph.), a disregard limit for any unknown impurity of 0.03 mg/V_max_ was suggested (Cardinale et al. 2017b). In this study, all impurities in a HPLC analysis range were integrated and counted as the sum of unknown impurities. This sum was at the same level as the disregard limit for a single unknown impurity. Thus, under correct application of the disregard limit, the produced [^18^F]PSMA-1007 batches can be considered as without impurities. The level of impurity meets the specification criteria for draft monographs. However, to ensure safety, since an injection solution synthesized using a CFN-MPS200, we conducted observational studies in mice using the decayed three-lot test solutions. In this study, we determined that the [^18^F]PSMA-1007 solution included the highest single chemical impurity (and non-radioactive PSMA-1007, other impurities and additives) without any adverse reaction observed. With respect to the tubing material, silicone essentially does not have high chemical resistance to organic solvents, but it was confirmed that MeCN, DMSO, and ethanol can be used only once. From these results, we evaluated silicone as the most suitable material for a [^18^F]PSMA-1007 synthesis cassette taking into account its cost. For the cassette with silicone tubings, the ACE salt precursor was compared against the TFA salt precursor, and the activity yields, the radiochemical yield, and the radiochemical purity were increased and all radiochemical impurities were reduced with the use of an ACE salt precursor. These results showed that the radiosynthesis of [^18^F]PSMA-1007 using the ACE salt precursor is superior to that with the TFA salt precursor in terms of radiochemical yield and radiochemical purity. One reason is that the ACE salt precursor was recrystallized from the TFA salt precursor; therefore, exhibiting a higher purity resulting in higher yields and lesser chemical impurities. In this study, we used a quaternary ammonium salt precursor without protective groups, but this radiosynthesis was performed with a very simplified and user-friendly method resulting in sufficiently high radiochemical yields.

The separation and purification of [^18^F]PSMA-1007 could be performed using the SPE method. Following radio-fluorination, the crude reaction mixture, diluted with an aqueous solution containing 5% EtOH, passed through the PS-H^+^ (L) and C_18ec_ (M) columns. The non-reacted precursor was trapped on the PS-H^+^ column as a cation exchange column and [^18^F] fluoride, water-soluble impurities, and organic solvents, among others, were eliminated by 23 mL of 5% EtOH. Further, remaining impurities on the C_18ec_ column were eluted by the first 3 mL of 30% EtOH. Finally, the second 4 mL 30% EtOH step eluted [^18^F]PSMA-1007 from the C_18ec_ column. We found that this ratio of 30% EtOH (3 and 4 mL) was reproducible for the separation and formulation of [^18^F]PSMA-1007 using the CFN-MPS200 synthesizer. It is critical to control the flow rate to obtain high reproducibility with the SPE method. The transfer of solution within the CFN-MPS200 synthesizer cassette is accomplished by a combination of N_2_ gas flow and vacuum pressure control. In this study, the loading and elution process, a particularly critical step, was performed with 50 mL/min flow of N_2_ gas, which was chosen in consideration of the control accuracy of the flow meter. We were able to perform the washing process over a short period while maintaining a constant flow rate using a combination of 50 mL/min of N_2_ gas and vacuum pressure. We believe that these conditions were a major factor in achieving the synthesis of [^18^F]PSMA-1007 with high reproducibility using CFN-MPS200 in this study.

## Conclusion

We have succeeded in the automated production of [^18^F]PSMA-1007 injection solution for clinical use with a new cassette and optimized the synthesis procedure using a CFN-MPS200 synthesizer. The radiosynthesis of [^18^F]PSMA-1007 solution, achieved via a one-step SPE method for separation and formulation, is very simple and stable. Furthermore, a washing / cleaning process of the synthesis module is not necessary because all materials used for the radiosynthesis are disposable. The availability of the cassette kit including reagents for CFN-MPS200 will facilitate the widespread use of [^18^F]PSMA-1007 PET and will help to better manage prostate cancer patients.

## Data Availability

The datasets used and/or analyzed during the current study are available from the corresponding author upon reasonable request.

## Abbreviations

ACE: Acetic acid
DMSO: Dimethyl sulfoxide
EOS: End of synthesis
HPLC: High-performance liquid chromatography
PET: Positron emission tomography
PSMA: Prostate-specific membrane antigen
SPE: Solid phase extraction
TBA: Tetrabutylammonium
TFA: Trifluoroacetic acid
WFI: Water for injection
RT: Retention time
